# Nutritional Practice and Nitrogen Balance in Elite Japanese Swimmers during a Training Camp

**DOI:** 10.3390/sports9020017

**Published:** 2021-01-21

**Authors:** Ami Mizugaki, Hiroyuki Kato, Haruka Suzuki, Hidefumi Kurihara, Futoshi Ogita

**Affiliations:** 1 Olympic and Paralympic Promotional Office, Corporate Service Division, Ajinomoto Co., Inc., 1-15-1 Kyobashi, Tokyo 104-0031, Japan; ami_mizugaki@ajinomoto.com (A.M.); haruka_suzuki@ajinomoto.com (H.S.); hidefumi_kurihara@ajinomoto.com (H.K.); 2 Department of Sports and Life Sciences, National Institute of Fitness and Sports, 1 Shiromizu-cho, Kanoya 891-2393, Japan; ogita@nifs-k.ac.jp

**Keywords:** nitrogen balance, elite swimmers, protein intake, leucine

## Abstract

The protein requirement in athletes increases as a result of exercise-induced changes in protein metabolism. In addition, the frequency, quantity, and quality (i.e., leucine content) of the protein intake modulates the protein metabolism. Thus, this study aimed to investigate whether nutritional practice (particularly, protein and amino acid intake at each eating occasion) meets the protein needs required to achieve zero nitrogen balance in elite swimmers during a training camp. Eight elite swimmers (age 21.9 ± 2.3 years, body weight 64.2 ± 7.1 kg, sex M:2 F:6) participated in a four-day study. The nitrogen balance was calculated from the dietary nitrogen intake and urinary nitrogen excretion. The amino acid intake was divided over six eating occasions. The nitrogen balance was found to be positive (6.7 ± 3.1 g N/day, *p* < 0.05) with protein intake of 2.96 ± 0.74 g/kg/day. The frequency and quantity of leucine and the protein intake were met within the recommended range established by the International Society of Sports Nutrition. Thus, a protein intake of 2.96 g/kg/day with a well-designated pattern (i.e., frequency throughout the day, as well as quantity and quality) of protein and amino acid intake may satisfy the increased need for protein in an elite swimmer.

## 1. Introduction

Dietary protein has essential roles in optimal exercise-induced adaptation and recovery from exercise [1]. An increased availability of amino acids increases the rate of muscle protein synthesis (MPS) synergistically with exercise [2], and leads to a positive net protein balance [3]. Endogenous oxidative loss must be replaced with a dietary source of amino acids in order to provide a substrate (like a building block) to repair and remodel the tissues during the recovery period [4]. As the need for amino acids increases after exercise, a higher protein consumption (1.2–2.0 g of protein/kg/day) is recommended for athletes compared with the current recommended daily allowance of protein (0.8 g/kg/day) [5]. However, as the need for amino acids during the recovery phase are modulated by the type or duration of exercise, as well as the training status of athletes [1,6,7,8,9], a pattern of protein consumption should be optimized according to the nature of exercise and the subjects’ characteristics.

In general, an athlete’s protein requirements are based on studies carried out in endurance exercise (i.e., running or cycling) [6,10,11] or resistance exercise [6,7,11]. Thus, few reports are available on the protein requirements for other types of sports. Swimming is a sport that demands technical skills and specific physical features (i.e., muscle power and endurance capacity with anaerobic and aerobic fuel systems). In general, swimmers undergo a high volume of training to improve their endurance capacity [12]. Moreover, dry-land exercises, weight-bearing strength training outside of the pool, and resistance training are incorporated into their training schedule to improve muscle power and to increase muscle mass [13,14,15]. However, metabolic demands during swimming dramatically vary according to stroke styles [16,17], skill levels [18], and water temperature [19]. Thus, special adjustments are needed to meet the nutritional needs after swimming, according to these different conditions.

In general, protein requirements are determined by the nitrogen balance (NBAL) technique [6]. A recent study using a current method, the indicator amino acid oxidation (IAAO) method, indicated that the NBAL technique may underestimate requirements [20]. However, as an isotopic steady state is required in the IAAO method, it is not applicable for determining protein requirements in the free-living state. Thus, to avoid interrupting the training schedule of an elite athlete, the NBAL technique is still the golden standard to quantify protein needs during a training camp. In the previous study [21], an average protein intake and a population-safe protein intake for achieving zero NBAL was found to be 1.4 and 1.9 g/kg/day in male competitive collegiate swimmers, respectively [21]. As an elite swimmer can swim more economically than non-elite swimmer [18], nutritional intervention for elite swimmers should be arranged according to their metabolic needs. However, there are limited reports relating the protein requirements in elite swimmers because of certain restrictions that are in place. For instance, participation in experimental studies may disturb the training schedule, while a limited number of elite athletes are available for participation. Thus, it remains unclear if the current recommendations meet the needs of elite swimmers.

Several recent consensus statements have reported that the frequency, quantity, and quality of protein should be considered in order to meet protein needs, as well as the total amount of daily protein intake [22,23]. Specifically, the frequency and quantity of the protein intake per eating occasion within a day affects the muscle protein metabolism [24,25]. Furthermore, MPS differs according to the type of ingested protein [26], primarily based on the amino acid content (especially leucine) [27]. According to recent consensus guidelines [22,23,28], 20–40 g of high-quality protein (0.25–0.40 g/kg/dose) or 10 g of essential amino acids (EAAs) should be consumed every 3 to 4 h in order to maximize post-exercise MPS for optimizing body composition and exercise performance. Furthermore, the consumption of 700–3000 mg of leucine or balanced EAAs enriched with leucine is recommended on each eating occasion [22]. It is, therefore, crucial to evaluate the dietary pattern (i.e., frequency throughout the day and composition of the amino acids ingested in each eating occasion) for determining the protein requirements.

Herein, we aimed to investigate the dietary protein intake and NBAL in elite swimmers during a training camp. Specifically, we examined the frequency, quantity, and quality of each eating occasion in order to clarify whether the pattern of protein intake meets the protein requirements.

## 2. Materials and Methods

### 2.1. Ethics Statement

Each subject received an explanation regarding the purpose of the study, experimental protocol, and all potential risks before taking part in this study. The experimental protocol was conducted in accordance with the Declaration of Helsinki, and was approved by the research ethics board of the National Institute of Fitness and Sports (8–61), as well as the institutional review board of Ajinomoto Co., Inc. (Tokyo, Japan) (2017-015). Subjects aged >20 years provided written informed consent, while in cases of subjects aged 18–19 years, both the subjects and their parents provided written informed consent. This trial was registered at www.umin.ac.jp/ctr/index.htm (UMIN 000029374).

### 2.2. Overall Experimental Protocol

**Figure 1:**
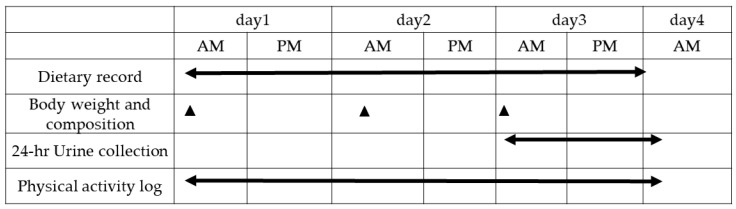
Study design.

The experiment was performed between December 2018 and January 2019 during subjects’ training camp, held at the Ajinomoto National Training Center. Swimmers who had competed in international competitions (such as the Olympic Games, Asian Games, FINA World Championships, and FINA World Championships (25 m)) as a member of the Japanese National Team, or who broke the qualifying standard for participation in Japanese national training camps, were selected for this study. The data collected from subjects who were unable to complete all of data collection were not included in the analysis. The experimental protocol is shown in Figure 1 and is consistent with previous work [21]. Briefly, following an overnight fast, the body weight, fat mass, and fat-free mass were measured using a bioelectrical impedance analysis (InBody 770, InBody Japan Inc., Tokoyo, Japan) throughout the experimental period. The date of the last menstrual period was obtained in order to predict whether the subjects were in the luteal or the follicular phase on the first day of experiment. The subjects ate breakfast, lunch, and dinner ad libitum in the dining room, located at the Ajinomoto National Training Center. Training activities were designed by the coaches, which contained dry-land exercises, swimming exercises, and resistance exercises. Dry-land exercises were weight-bearing strength exercises to improve muscle strength. Resistance exercises comprised of whole-body resistance exercises, including squats, bench press, and leg press. On day 3, to quantify nitrogen excretion, urine was collected continuously for 24 h following the first urination, up until the first urination on the following morning. Subjects wore an accelerometer (wGT3X-BT, Actigraph, Pensacola, FL, USA) during the study period (excluding bathing and exercise) to assess their normal daily physical activity.

### 2.3. Dietary Intake

On days 1, 2, and 3, all of the dietary intake for each subject was assessed by a registered dietitian, as previously described [29]. Three main meals (breakfast, lunch, and dinner) were provided at the buffet-style dining hall. Subjects were asked to keep their usual eating habits. Nearly all of the dishes on the menu, except for steamed rice, were served in standardized sizes. The steamed rice was weighed using a scale. A registered dietician checked the food items selected and recorded the leftovers after each meal. Subjects were allowed to purchase any foods from outside of the restaurant as snacks. All snacks (food, fruit, supplements, and beverages) were recorded during the experimental period with detailed information (e.g., weight or sizes, brand names, and time of consumption). Based on the assessment, the dietary intake (energy, carbohydrates, proteins, amino acids, and fats) was calculated using a software (Excel Eiyo-kun ver. 8 (Kenpaku-sha, Tokyo, Japan)) that calculates the content of nutrients in foods. To analyze the quantity and quality (i.e., amino acid composition) of protein intake per eating occasion throughout the day, the dietary intake was divided into six eating occasions, namely: three main meals (i.e., breakfast, lunch, and dinner) and three snacks (morning snack, afternoon snack, and pre-sleep snack).

### 2.4. Energy Expenditure and Energy Balance

The total daily energy expenditure was estimated by adding the resting energy expenditure (REE), energy expenditure during normal daily physical activities, diet-induced thermogenesis (DIT), and exercise-induced energy expenditure (EEE) values. The REE values were calculated using Nelson’s equation, as follows: REE (kcal/day) = 25.80 × fat-free mass (kg) + 4.04 × fat mass (kg) [30]. Energy expenditure induced by normal daily physical activities were calculated from the data of an accerelometer they wore. The DIT was estimated from the total energy intake multiplied by the ratio of DIT to total energy reported in a previous study [31], as the protein–fat–carbohydrate balance of the consumed meals in this study was similar to that found in the previous study. A heart rate monitor (M430, Polar Electro, Kempele, Finland) was worn during swimming and dry-land exercise sessions to measure the quantity and intensity of each exercise. The EEE during swimming and dry-land exercises was assessed using the average heart rate (HR), exercise duration (time), gender, body weight, and age using Keytel’s equation [32]. In particular, the EEE during resistance exercises was calculated from the reps and duration of exercises according to Phillips’ study [33]. The energy balance between the energy intake and total energy expenditure was calculated each day.

### 2.5. Nitrogen Balance

The urine samples were stored at 4 °C until analysis after acidification with anhydrous citric acid. The creatinine and urea concentrations in the urine were measured by SRL Medisearch, Inc. (Shinjuku, Tokyo, Japan). Nitrogen excretion was calculated by summing the urinary excretion of urea-bound nitrogen and creatinine-bound nitrogen, which represents >75% of the total nitrogen excretion in athletes [11]. NBAL was estimated as follows: NBAL = E − I, where E represents nitrogen excretion and I represents dietary nitrogen intake, calculated as the intake of protein and amino acids on day 3 divided by a nitrogen-to-protein conversion factor (6.25).

### 2.6. Data Analyses

Values are reported as the mean ± standard deviation or a 95% CI range. Paired t-tests were used to compare the body weight and body composition between day 1 and 4, and whether the energy balance on day 1, 2, and 3, or the NBAL on day 3 was positive or negative. Linear regression analyses were applied to determine the relationship between the protein intake and the NBAL. Data were analyzed using the GraphPad Prism 6 software (GraphPad Software Inc., San Diego, CA, USA); *p* < 0.05 was considered significant.

## 3. Results

### 3.1. Subjects Characteristics

We aimed to recruit ten subjects. However, one subject could not participate in this study because of their training schedule. Thus, we enrolled nine subjects. In addition, the data from one subject was excluded from analysis as a result of failure to collect a urine sample. Thus, eight subjects completed the study and were included in the analysis. The characteristics of the subjects are presented in Table 1. No significant changes were observed in body weight, fat mass (%, kg), or fat-free mass over the experimental period (*p* > 0.05, Table 2).

### 3.2. Dietary Intake

**Figure 2:**
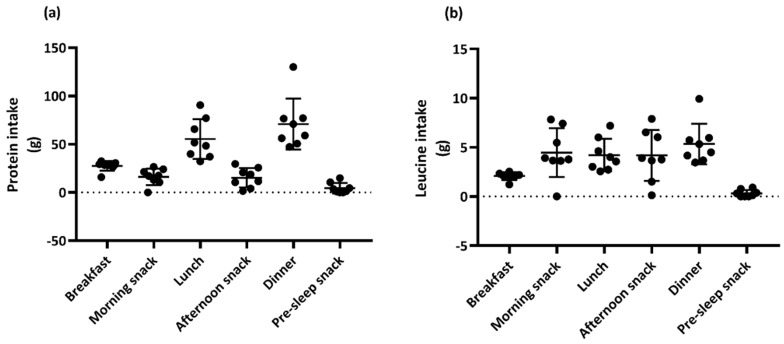
Amount of dietary protein and leucine consumed in each eating occasion on day 3. Protein intake in each meal or snack (**a**), and leucine intake (**b**). Data are shown as mean ± standard deviation (*n* = 8). *: significant difference between the lower end of the recommended intake (20 g for protein and 0.7 g for leucine) and the actual intake by subjects [22,23].

The data on the dietary intake from days 1, 2, and 3 are shown in Table 3. The protein intake on day 3 was 3.0 ± 0.7 g/kg/day. The quantity of the dietary protein and leucine consumed in each meal or in the snacks on day 3 is shown in Figure 2. The protein intake in the meals (i.e., breakfast, lunch, and dinner) was 27.5 ± 5.0, 55.4 ± 20.6, and 71.0 ± 26.5 g/meal, respectively. The leucine intake in the meals was 2.1 ± 0.4, 4.2 ± 1.7, and 5.3 ± 2.1 g/meal, respectively. The protein intake in the morning, afternoon, and pre-sleep snacks was 16.2 ± 8.5, 15.3 ± 10.1, and 4.5 ± 5.5 g/snack, respectively. The leucine in the three snacks accounted for 4.5 ± 2.5, 4.2 ± 2.6, and 0.3 ± 0.4 g/snack, respectively.

### 3.3. Energy Expenditure

The volume (duration and covered distance) and intensity of each exercise session during the 3 days are shown in Table 4. No. 7 did not swim on day 1, and No. 8 did not swim on day 1 in the p.m. and day 2 in the a.m. No. 7 and 8 also missed their resistance exercise session on day 2. The energy expenditure during the resistance exercises on Day 2 was calculated as 291.5 ± 51.5 (*n* = 6). The total daily energy expenditure with the REE, DIT, and EEE (normal daily physical activity and training sessions) values, are shown in Table 5.

### 3.4. Energy Balance

**Figure 3:**
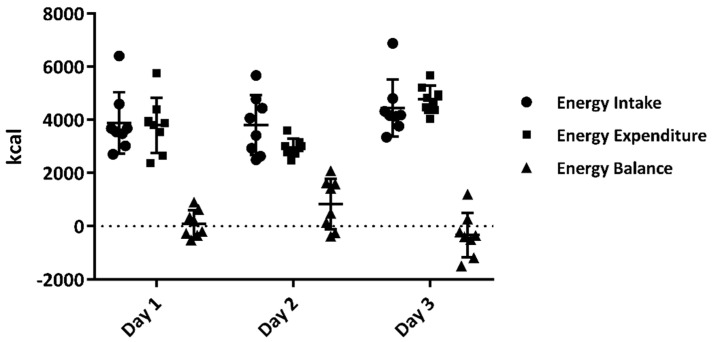
Energy balance (energy intake and energy expenditure) during the experimental period. Data are shown as mean ± standard deviation (*n* = 8).

The energy balance was 96 ± 506, 646 ± 975, and -333 ± 830 on days 1, 2, and 3, respectively (Figure 3). There were no significant differences compared to zero on days 1, 2, and 3 (*p* > 0.05, for all).

### 3.5. Nitrogen Balance

The protein intake and NBAL of each subject is summarized in Table 6. No linear relationship was observed between the protein intake on day 3 and NBAL (R^2^ = 0.003, *p* > 0.89, *n* = 8). The mean NBAL was significantly higher than zero (*p* < 0.05).

## 4. Discussion

In this study, we determined the NBAL in elite swimmers by analyzing the dietary pattern of the protein and amino acid intake throughout the day. The average protein intake was 2.96 g/kg/day and the protein intakes at breakfast, lunch, and dinner were higher than the recommended protein intake (20–40 g) for maximizing the MPS. Furthermore, the leucine intake met the recommended dose (700–3000 mg) in all meals and snacks, except in the pre-sleep snack. Importantly, NBAL was significantly higher than zero balance. Thus, 2.96 g/kg/day of average protein intake with an optimal protein quality (i.e., leucine content) in five eating occasions and an optimal protein quantity in three main meals surpassed the protein needs in elite swimmers during the training camp.

According to the ACSM position stand, 1.2–2.0 g/kg/day protein intake is recommended for active and athletic populations [1]. In our previous study, average protein requirements in male competitive collegiate swimmers, for a zero-nitrogen balance and a population-safe protein intake, was reported to be 1.4 and 1.9 g/kg/day, respectively [21]. However, certain differences were noted between this previous study and the current one, including training volume, characteristics of subjects, and nutritional intake, which potentially affected the protein requirements [21]. First, our subjects trained for 6 h/day and then swam 12,000 m/day, while the competitive collegiate swimmers in our previous study trained for 6 h/day with 10,000 m/day swim sessions [21]. As the training volume increases, the oxidative loss of amino acid also increases, and hence, the protein needs after exercises in this study might have increased. However, elite swimmers can swim more economically compared with non-elite swimmers [20]. Thus, the difference of the training volume might be trivial. Second, there are differences in the characteristics (i.e., training status) of the subjects. In the current study, the protein needs might have decreased because of the training status of the subjects. We recruited Japanese elite swimmers, who either had competed internationally as a member of the Japanese National Team or who had surpassed the qualifying standard for participation in a Japanese national training camp. Although we did not measure the VO_2max_ of elite swimmers in this study, it was reported to be as 72–76 mL/kg/min [34]. However, the VO_2max_ of the competitive collegiate swimmers in our previous study was 63.9 mg/kg/min [21]. Endurance training suppresses oxidative loss of amino acid during exercises [35], and resistance training utilizes protein efficiently by altering the protein metabolism [36,37]. Third, other nutritional statuses might affect protein metabolism. While the subjects in the previous study showed a significant negative energy balance due to insufficient energy intake, the subjects in this study showed a zero-energy balance on the day of urine collection. A negative energy balance leads to increased whole-body catabolism, increased oxidative loss of amino acids, and increased excretion of nitrogen [38,39], which may further result in increased protein requirements [22,40,41]. In addition, in our previous study, the subjects did not meet the recommended carbohydrate intake during the high volume training phase [1]. An insufficient availability of carbohydrates causes an increase in the oxidative loss of endogenous proteins [42,43] and decreases the MPS [43]. In the current study, all subjects had a positive energy balance and a higher carbohydrate intake than recommended. Lastly, the sex of the participants might affect the protein requirements. While male participants were recruited in the previous study, 75% of the participants were females in the current study. Menstrual cycle or estrogen levels affect amino acids’ metabolism [44,45]. While estrogen decreased the whole body leucine oxidation [45], urinary nitrogen excretion was high during the luteal phase, when estrogen and progesterone were high [46]. Consequently, the lysine requirements [45], leucine flux, and oxidation [47] were elevated during the luteal phase. According to previous studies, it has been reported that there is a difference in the protein requirements between male and female athletes after resistance exercises [9,48] and variable-intensity exercise [49,50]. To summarize, although there were potential factors affecting the protein needs, 2.96 g of protein/kg/day was sufficient to induce a positive NBAL. However, as there was no linear relationship between the protein intake and the NBAL in the current study, it was not possible to establish a correlation between protein intake and population-safe intake. In general, the population-safe intake was set based on a statistical model to ensure the protein needs in approximately 98% of the population, accounting for individual differences. The population-safe intake guidelines were used as the recommended protein intake. In our study, all subjects showed a positive NBAL. The lowest protein intake (2.3 g/kg/day) in our subjects might be assumed to ensure the protein requirement in this situation. Further studies are needed to assess the protein requirement and population-safe protein intake for levels above 2.3 g protein/kg/day.

The protein intake for the three main meals met the recommended protein intake (20–40 g) for maximizing the MPS [2,51,52], and the leucine intake also met the provided guidelines [22,23] in all meals and snacks, except the pre-sleep snack. Unlike in our previous study [21], the leucine intake was sufficient for meeting the recommended dose of leucine in the morning and afternoon snacks in the current study, as the subjects consumed supplemental foods containing amino acids, especially EAA enriched with leucine, immediately after their training sessions. According to the previous report, an egg protein-based diet enriched with leucine, isoleucine, and valine decreases the requirements of amino acids in endurance athletes [53]. Thus, the supplemental consumption of leucine-enriched EAA between meals might help to meet the increased needs for amino acids. Nevertheless, the subjects consumed a pre-sleep snack. As overnight sleep is typically the longest fasting period, protein ingestion immediately prior to sleep is recognized as a beneficial way to increase MPS [54]. In the current study, the subjects consumed a large portion of protein at dinner, approximately 2 h before sleep. As the protein ingested at dinner was much higher than the required dose (i.e., 40 g), to maximize the MPS, ingesting protein at dinner and pre-sleep snack might be a more beneficial way of optimizing protein metabolism overnight during sleep. Future studies are needed in order to determine the effect of snacks containing high quality amino acids and pre-sleep ingestion on protein requirements.

Taken together, the results of the current study suggest that adopting a protein and amino acid intake pattern containing an adequate quantity of 2.3 g of protein/kg/day (20–40 g of protein/meal) in three main meals, as well as two additional snacks, including an adequate quality (700–3000 mg of leucine intake per snack), can meet the protein needs of elite swimmers during training camps in order to achieve a positive NBAL. The data from this study reveal some practical applications worthy of future study. Considering these results, proper snacks containing sufficient leucine might effectively avoid the need for increasing the protein consumption from meals. Moreover, diets containing sufficient amounts of energy to meet the increased energy expenditure associated with high volume training, at a normal ratio of protein, fat, and carbohydrates, can easily meet the 2.3 g of protein/kg/day. For example, in cases when ideal diets are not attainable from meals alone, because of the special circumstances associated with training camps, portable snacks or supplements might help to meet the protein needs for achieving a positive NBAL.

Nevertheless, certain limitations were noted in this study. First, to avoid the common challenges associated with conducting experiments on elite athletes, the current study was designed as an observational study with a limited number of subjects. Because of the small number of subjects, there may be potential limitations in applying the results to all elite swimmers. However, as the training and the trained status of every elite athlete is assumed to be specific, there is an inherent difficulty in attempting to define representative values for all elite athletes. Thus, it is important to assess the nutritional status and protein metabolism in each situation. Second, to set the protein requirements and population-safe intake standards, using the NBAL technique, both the lower and upper values of the actual protein requirement were needed to apply the linear regression analysis. In the current study, the subjects consumed their diet *ad libitum* in the dining room at the Ajinomoto National Training Center, which provides a high quality and sufficient diet. As a result, they consumed a sufficient protein intake (95% CI: 2.3–3.6 g/kg/day). Taking all meals into account, 2.3 g/kg/day might be sufficient to induce a positive NBAL in the current study. In addition, we measured the protein intake and NBAL on day 3 only. Because of some potential variation (i.e., dietary intake, content of training sessions, and so on), it might lead to making some errors in the NBAL measurements for determining the protein requirements. Therefore, further interventional studies might shed light on the protein requirements needed for maintaining NBAL in elite swimmers during training camps. Third, although we utilized the NBAL technique to assess the protein metabolism by considering the amount and daily pattern of the protein intake; the protein requirements should be determined as per body composition and/or exercise performance. In addition, a recent study reported that the protein requirements determined using the indicator amino acid oxidation (IAAO) method were higher than the protein requirements determined with the NBAL technique [8,10]. The results indicated that the NBAL technique may underestimate the true requirements [20]. In addition, the relationship between NBAL and exercise performance is equivocal. In a recent report, a protein intake that induced a positive net balance indicated a better exercise performance compared with a protein intake maintaining a zero net protein balance [55]. Thus, further studies are needed to confirm if the protein intake is optimal for optimizing exercise performance in elite swimmers during their training camps.

## 5. Conclusions

In conclusion, NBAL was significantly positive in Japanese elite swimmers during a training camp, when they had an average protein intake of 2.96 g/kg/day (95% CI range: 2.34–3.57) with an optimal frequency, quantity, and quality of protein, including meals and snacks throughout the day. Thus, the lower end of the 95% protein intake (2.3 g/kg/day) with an optimal pattern of protein and leucine intake may satisfy the protein requirements for positive NBAL during a training camp in an elite swimmer. Further investigations are warranted in order to determine the pattern of the protein and amino acid intake for optimizing exercise performance.

## Figures and Tables

**Table 1:** Characteristics of the subjects at the beginning of the study.

Characteristic	Overall	Male	Female
Age (yrs)	21.9 ± 2.3	24.0 ± 0.0	21.2 ± 2.2
Height (cm)	170.9 ± 5.9	176 ± 2.8	169.2 ± 5.8
Body weight (kg)	64.2 ± 7.1	74.8 ± 2.8	60.6 ± 2.9
Fat free mass (kg)	53.7± 7.4	65.4 ± 0.1	49.9 ± 1.9
Body fat (%)	16.4 ± 4.4	12.5 ± 3.4	17.7 ± 4
N	8	2	6

Data are shown as mean ± SD.

**Table 2:** Changes in body weight and body composition.

Variable	Day 1	Day 4	*p* Value
Body weight, kg	64.2 ± 7.0	64.2 ± 7.2	n.s
Fat mass, %	16.4 ± 4.5	16.3 ± 4.2	n.s
Fat mass, kg	10.5 ± 2.9	10.4 ± 2.6	n.s
Fat-free mass, kg	53.7 ± 7.3	53.8 ± 7.4	n.s

Data are shown as mean ± standard deviation (*n* = 8). n.s, not significant.

**Table 3:** Daily total energy and macro-nutrient intake.

Variable	Day 1	Day 2	Day 3 (Test Day)
Energy			
kcal/day	3889 ± 1156	3803 ± 1128	4447 ± 1068
Protein			
g/day	166 ± 57	173 ± 49	190 ± 56
g/kg/day	2.6 ± 0.8	2.7 ± 0.7	3.0 ± 0.7
Energy %	17 ± 2	18 ± 1	17 ± 1
Fat			
g/day	117.6 ± 23.2	110.2 ± 32.2	136.3 ± 35.7
g/kg/day	1.8 ± 0.4	1.7 ± 0.5	2.1 ± 0.5
Energy %	28 ± 4.4	26.2 ± 2.9	27.6 ± 2.8
Carbohydrate			
g/day	530 ± 208	531 ± 166	614 ± 136
g/kg/day	8.3 ± 3.1	8.2 ± 2.3	9.6 ± 1.9
Energy %	53 ± 7	56 ± 3	56 ± 4

**Table 4:** The volume and intensity of exercise sessions during the experimental period.

Day	Variable	Morning Sessions		Afternoon Sessions	
		Dry-Land	Swimming	Dry-Land	Swimming
Day 1	HR, bpm	95.2 ± 9.0	124.8 ± 4.9	86.8 ± 6.1	119.6 ± 5.1
	Time, min	58.8 ± 10.0	143.0 ± 21.6	55.1 ± 1.7	148.4 ± 2.7
	Distance, m		5886 ± 567		6833 ± 408
Day 2	HR, bpm	97.7 ± 15.0	139.3 ± 6.1		
	Time, min	51.3 ± 9.4	140.4 ± 3.9		
	Distance, m		6441 ± 2777		
Day 3	HR, bpm	89.6 ± 7.9	126.6 ± 6.3	93.3 ± 4.6	122.2 ± 4.9
(Test day)	Time, min	57.1 ± 13.9	156.8 ± 22.6	56.1 ± 13.6	120.9 ± 18.7
	Distance, m		6844 ± 515		5630 ± 765

Data are shown as mean ± standard deviation (*n* = 6–8). HR—the average heart rate (bpm) during exercise session; time—duration (in minutes) of the exercise session; distance—distance (m) subjects covered during the swimming exercise session.

**Table 5:** Energy expenditure during the experimental period.

kcal/Day	Day 1	Day 2	Day 3
Resting energy expenditure	1429 ± 187		
Diet-induced thermogenesis	276 ± 82	270 ± 80	316 ± 76
Exercise-induced energy expenditure			
Normal daily physical activity	345 ± 97	319 ± 68	252 ± 98
Exercise sessions	1742 ± 967	1139 ± 286	2783 ± 497
Total energy expenditure	3792 ± 1048	3157 ± 429	4780 ± 509

Data are shown as mean ± standard deviation (*n* = 8). Diet-induced thermogenesis was calculated from the total energy intake times 7.1% [26].

**Table 6:** Summary of protein intake and nitrogen balance.

Subject No.	Protein Intake (g/kg/day)	Nitrogen Balance (g N/day)
1	3.59	0.47
2	4.39	10.34
3	3.16	6.76
4	2.57	8.32
5	2.42	9.46
6	2.25	5.77
7	2.37	4.73
8	2.89	7.96
mean (95% CI range)	2.96 (2.34–3.57)	6.73 (4.11–9.34)

## Data Availability

The data presented in this study are available on request from the corresponding author.
